# Islands as a crossroad of evolutionary lineages: A case study of *Centaurea* sect. *Centaurea* (Compositae) from Sardinia (Mediterranean Basin)

**DOI:** 10.1371/journal.pone.0228776

**Published:** 2020-02-07

**Authors:** Javier López-Alvarado, Giulia Mameli, Emmanuele Farris, Alfonso Susanna, Rossella Filigheddu, Núria Garcia-Jacas

**Affiliations:** 1 Systematics and Evolution of Vascular Plants (UAB)–Associated Unit to CSIC, Unitat de Botànica, Departament de Biologia Animal, Biologia Vegetal i Ecologia, Facultat de Biociències, Universitat Autònoma de Barcelona, Bellaterra, Spain; 2 Dipartimento di Chimica e Farmacia, Università degli Studi di Sassari, Sassari, Italy; 3 Institut Botànic de Barcelona (IBB, CSIC-ICUB), Barcelona, Spain; University of Florida, UNITED STATES

## Abstract

The Mediterranean Basin is a biodiversity hotspot, where islands play a key role because of their high biological diversity, degree of endemicity and human pressure. One of these islands, Sardinia, is a good evolutionary laboratory, especially for the study of complex genera, such as *Centaurea*. In particular, endemic species of *Centaurea* sect. *Centaurea* from Sardinia provides an interesting case study of plant evolution on continental islands. We attempted to clarify the processes leading to the diversification of *Centaurea* species on Sardinia using bi-parentally inherited nuclear markers and maternally inherited plastid markers. Our plastid results revealed the presence of five lineages of sect. *Centaurea* on the island. Three of them were defined as three species: *C*. *ferulacea*, *C*. *filiformis* and *C*. *horrida*. The other two lineages highlighted the complex evolutionary history of the two polyploids *C*. *corensis* and *C*. *magistrorum*. Multiple colonization events from the mainland involving the *C*. *deusta* and *C*. *paniculata* lineages among others, have led to the diversity of sect. *Centaurea* on Sardinia. One colonization event likely followed a southern path via the land connection between the mainland, the Calabrian Plate and Sardinia. A second pathway likely followed a northern connection, probably through the Tuscan Archipelago. Implications of these findings on conservation efforts for *Centaurea* endemics on Sardinia are also discussed.

## Introduction

The Mediterranean Basin has been considered a biodiversity hotspot because of its high degree of biological diversity, endemicity and human pressure [1, 2]. It contains approximately 4.3% of the world’s vascular plants and the level of endemicity is 52% [3]. Islands play important roles within the Mediterranean Basin, because four of the ten hotspots (Tyrrhenian islands, S. & C. Greece, Crete, S. Anatolia, and Cyprus) are themselves islands or include islands [2]. The Tyrrhenian islands include the second largest in the Mediterranean, Sardinia, which is an ecologically diverse and endemism-rich hotspot with 2149 native plant taxa displaying 13.5% endemism [4]. The Sardinian-Corsican biogeographic province comprises 260 endemic taxa [5], including three genera, namely *Castroviejoa* Galbany, L. Sáez & Benedí, *Morisia* J. Gay, and *Nananthea* DC.

As a continental island (sensu [6]) with several connections and disconnections with the mainland during its history [7–12], Sardinia is a good evolutionary laboratory. It shares a complex history with the rest of the region, but its insularity allows for research on geographically well-defined taxa. Consequently, we can avoid most of the taxonomic complexity and blurred boundaries that exist among taxa occurring on the mainland. Because of long-term isolation, one would expect to find only a small degree of genetic variation; thus, facilitating the tracing of evolutionary relationships ([13], but see [14] and references therein). However, genetic drift and other population genetic processes can make it difficult to reconstruct true phylogenetic relations [15].

The endemic species of the genus *Centaurea* L. on Sardinia provide an interesting case study of plant evolution on continental islands that may help to clarify the biogeographical history of Sardinia. *Centaurea* is a classic example of complex evolution, in that it involved hybridization and reticulate evolution, recognized in several sections and species of the genus (e.g., [16–18]). *Centaurea* consist of annual, biennial, or perennial herbs, rarely shrubs, often with unarmed leaves [19]. Based on morphology, pollen types, and phylogenetic studies [20–22], recent revisions accept three subgenera, namely *Lopholoma*, *Cyanus*, and *Centaurea*, with the latter being the most species-rich and divided into several sections [23]. Species of section *Centaurea* are distributed around the Mediterranean and are characterized by a basic chromosome number of *x* = 9 and deeply divided leaves [23]. Species of this section are not fully separated by intrinsic breeding barriers and hybrids are frequent [24–26], often homoploid and fertile [27–30], and sometimes polyploid [31, 32].

The endemic species in Sardinia constitute a clearly delimited group composed of only five species and one nothospecies: *Centaurea corensis* Vals. & Filigh., *C*. *ferulacea* Martelli, *C*. *filiformis* Viv., *C*. ×*forsythiana* Levier, *C*. *horrida* Badarò, and the extremely narrow endemic *C*. *magistrorum* Arrigoni & Camarda, all of which belong to the subgenus *Centaurea* sect. *Centaurea* and are classified either in subsect. *Centaurea* with spiny-tipped, pectinate-fimbriate appendages, or in subsect. *Phalolepis* with unarmed, membranaceous, lacerate appendages [33]. The remaining species of *Centaurea* that grow in Sardinia are widespread and belong to unrelated sections or subgenera, and although hybrids have been reported between species that are not closely related [34–36], they are rare, do not produce fertile offspring, and do not contribute to reticulation.

*Centaurea corensis* is a perennial tetraploid (2*n* = 36 [37]) with a woody base, leaves in a basal rosette, white to pale pink capitula, and membranaceous bract appendages. The species grows only at one site in northwestern Sardinia and is morphologically similar to the *C*. *deusta* Ten. aggregate from the mainland. *Centaurea ferulacea*, *C*. *filiformis* and *C*. *horrida* are long-living perennial diploids with 2*n* = 18 [29, 38–40]. *Centaurea horrida* is a pulvinular chamaephyte with spiny leaves and solitary capitula with shortly fimbriate bract appendages, growing exclusively on coastal cliffs, forming part of the dwarf vegetation of northwestern Sardinia (with a single remote isolated population on the islet of Tavolara, northeasten Sardinia). *Centaurea filiformis* and *C*. *ferulacea* are both chasmophytic, unarmed perennials living on limestone cliffs in eastern Sardinia (Supramontes, Gulf of Orosei, Tavolara islet). They differ mainly in the involucral bracts of their capitula, being black and pinnatifid with a small terminal spine in *C*. *filiformis*, as in other species of subsect. *Centaurea*, and straw-colored, hyaline, lacerate-fimbriate, unarmed in *C*. *ferulacea*, as in species of subsect. *Phalolepis*. *Centaurea magistrorum* is a perennial triploid with *x* = 9 (R. Filigheddu & G. Becca, unpublished research) and is morphologically similar to the *C*. *paniculata* L. complex from the mainland, with erect stems and multiple capitula with shortly fimbriate bract appendages [41]. It grows on granitic soils and is known only from the type locality. Finally, the nothospecies *C*. ×*forsythiana* is a long-established homoploid hybrid between *C*. *filiformis* and *C*. *horrida* from the Tavolara islet [42].

In this context, we attempted to elucidate the processes leading to the diversification of *Centaurea* section *Centaurea* in Sardinia using bi-parentally inherited nuclear markers and maternally inherited plastid markers analyzed with phylogenetic inference and networking methods. Our specific goals were to: 1) investigate the evolution and biogeography of section *Centaurea* in Sardinia; 2) test the correspondence of traditional taxonomic entities and evolutionary lineages; 3) clarify the evolutionary history of the most enigmatic of the five Sardinian endemics, the narrow endemic *C*. *magistrorum*; and 4) address conservation efforts for this group of species.

## Materials and methods

### Plant material

**Fig 1:**
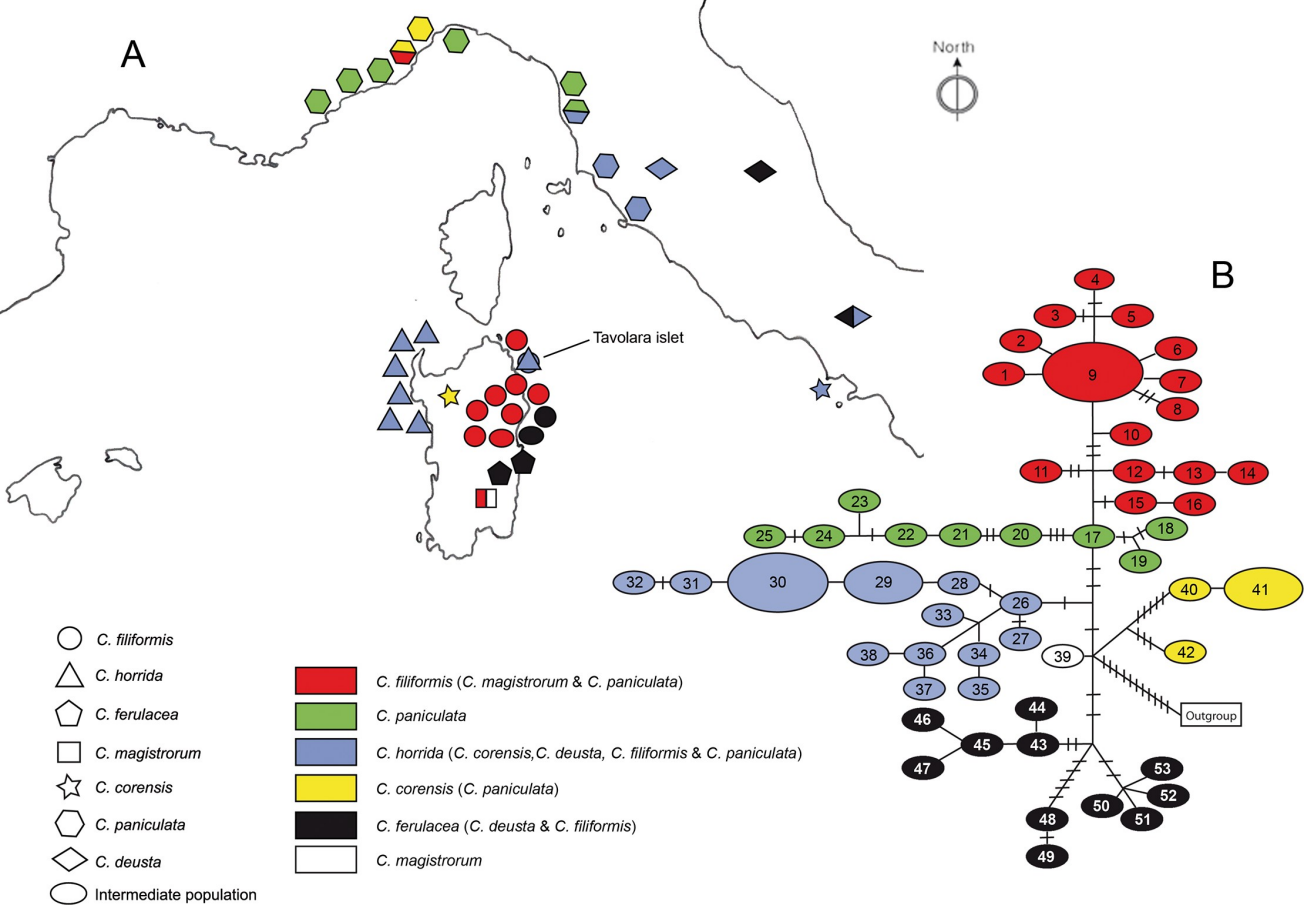
ptDNA parsimony network. **A,** Location of sampled populations for each species coded as different geometrical forms, haplotypes for each species, and populations coded with different colors as indicated in the legend. **B,** Haplotype network obtained by analysis of ptDNA markers *rpl16*, *rpl32-trnL*^*UAG*^, *rps4-trnT-trnL*, *trnG* and *ycf3-trnS*; numbers relate each haplotype to a species and population (S1 Table).

Collection of plant material was allowed by the Regione Autonoma della Sardegna and the Marina Militare Italiana. We sampled 125 individuals from 36 populations belonging to eight different species, the six Sardinian endemics, including *C*. ×*forsythiana*, and two widespread and clearly morphologically related species from the mainland, namely *C*. *deusta* and *C*. *paniculata*. We sampled two to five individuals from each population. For *Centaurea horrida*, 20 individuals were collected in seven populations from the entire range of the species. For *Centaurea filiformis*, we sampled 40 individuals from 12 populations covering almost all the entire range of the species. Two populations at Cala Sisine and Monte Oseli were morphologically intermediate between *C*. *filiformis* and *C*. *ferulacea* [43]. For *C*. ×*forsythiana*, we sampled three individuals from the only known population on Tavolara islet, which were used for the nrDNA analysis. For *C*. *ferulacea*, seven individuals from two populations were sampled, representing the entire range of the species. Finally, six individuals respectively were collected from the only known locality of *C*. *magistrorum* and *C*. *corensis* in Sardinia. We also added five individuals from a recently discovered population of *C*. *corensis* in Procida Island (Naples, Italy). Species from the mainland were included; namely, members of the *C*. *paniculata* complex, which are morphologically close to *C*. *filiformis* and *C*. *magistrorum* (cf. [44]), and *C*. *deusta*, morphologically closer to *C*. *ferulacea* and *C*. *corensis*. Sampling for *C*. *paniculata* consisted of 29 individuals from 10 populations ranging from southern France to central Italy. Sampling for *C*. *deusta* covered most of the area in the center of continental Italy. The geographical distribution of sampling is illustrated in Fig 1A. Furthermore, two species from the *Jacea-Phrygia* group [45] were selected as outgroup taxa. The voucher and GenBank accession numbers are given in S1 Table.

### DNA extraction, amplification, and sequencing

Each sample of field-collected leaf tissue was kept on ice or directly frozen in the field in liquid nitrogen. Total genomic DNA was obtained by grinding frozen leaves (approximately 100 mg) in a mortar containing liquid nitrogen and extracting and purifying the DNA using a DNeasy Plant Mini Kit (Qiagen, Italy) according to the manufacturer’s instructions. The average concentration of the extracted DNA obtained was 20 ng/μL (checked with a Nanodrop ND 1000 apparatus, ThermoFisher Scientific, Wilmington, DE, USA).

We selected the nrDNA internal transcribed spacer (ITS; Table 1), which has been frequently employed, to detect copies acquired by hybridization events, because concerted evolution is usually incomplete in *Centaurea* ([46] and references therein). For plastid markers (Table 1), we selected five loci: *rpl16*, *rpl32-trnL*^*UAG*^, *rps4-trnT-trnL*, *trnG*, and *ycf3-trnS*.

**Table 1:** Selected regions of nrDNA and ptDNA, including forward and reverse primers used for amplification.

Locus	Forward primer	Reverse primer	Reference
ITS	17SE	26SE	[47]
*rps4-trnT-trnL*	rps4R2	trnL-b	[48, 49]
*rpL16*	rpL16F71	RexC	[50, 51]
*ycf3-trnS*	SP43122F	SP44097R	[52]
*trnG*	3′trnG^UUC^	5′trnG2G	[48]
*rpl32-trnL*^*UAG*^	rpl32F	trnL^(UAG)^	[53]

nrDNA from the ITS region was amplified following a previously described protocol [45]. The PCR products of the ITS region for one individual from each of the Sardinian populations, as well as the Procida population of *C*. *corensis* were cloned with a TOPO TA Cloning Kit (Invitrogen, Carlsbad, CA, USA) following the manufacturer’s instructions. When possible, eight to 16 positive colonies from each reaction were screened using T7 and M13R universal primers, following a previously described amplification profile [54]. For ptDNA, the intergenic spacer *rps4-trnT-trnL*, the *rpL16* intron, the intergenic spacer *ycf3-trnS*, the *trnG* intron, and the *rpl32-trnL*^*UAG*^ intergenic spacer were amplified following a previously described protocol [46].

The PCR products were purified using ExoSAP-IT (USB Corp., Cleveland, OH, USA) and a QIAquick PCR Purification Kit (Qiagen Inc., Valencia, CA, USA). Direct sequencing of the amplified DNA segments was performed following the manufacturer’s protocol and using the BigDye Terminator Cycle Sequencing v3.1 (Applied Biosystems) at the University of Florida ICBR Core Facility using an ABI 3730xl (Applied Biosystems) and at Macrogen Inc., Korea.

Nucleotide sequences were edited using BIOEDIT v7.0.5.3 [55] and were aligned manually by sequential pairwise comparison [56]. Unique substitutions in clones from a single accession were excluded. Consensus sequences were generated for each accession with the goal of reducing redundant cloned sequences; thus, reducing matrix size and the effects of PCR artifacts (e.g., chimeric sequences and Taq errors [57, 58]).

### Phylogenetic analyses

Maximum parsimony (MP) and Bayesian inference (BI) analyses were conducted on the ITS and ptDNA datasets. Two species from the *Jacea-Phrygia* group, *C*. *emigrantis* and *C*. *subtilis*, were selected as the outgroup.

BI estimation was calculated using MrBayes 3.2 [59]. The model of molecular evolution was selected using the Akaike information criteria (AIC) and Bayesian information criterion (BIC) with jModeltest 0.1.1 [60, 61]. For the ITS and ptDNA alignments, the symmetrical model with variable base frequencies and gamma-distributed rate heterogeneity (GTR+G) was selected as the best-fit model of nucleotide substitution [62, 63]. BI analyses were initiated with random starting trees and runs for 20 ×10^6^ generations. Four Markov Chains were run using Markov Chain Monte Carlo (MCMC) principle sample trees. We saved one out of every 1000 generations, which resulted in 20000 sample trees. Data from the first 5000 generations were discarded as “burn-in”, after we had confirmed that the likelihood values had stabilized prior to the 5000^th^ generation. The convergence of MCMC chains (ESS>200) was checked using TRACER [64]. Posterior probabilities ≥ 95% were considered significant. The resulting tree was visualized using FigTree 1.4.3 [65].

MP analyses involved heuristic searches conducted with PAUP* version 4.0b10 [66] using tree-bisection-reconnection (TBR) branch swapping with character states specified as unordered and unweighted. All of the most parsimonious trees (MPTs) were saved. To locate other potential islands of MPTs [67], we performed 1000 replications with random taxon addition. The strict consensus of MPTs was calculated (tree not shown). Bootstrap analysis followed the approach by Lidén et al. [68] using 1000 replicates, random taxon addition with 100 replicates, and TBR no branch swapping. Bootstrap support values ≥ 70% were considered significant.

Networking analysis was conducted to visualize character incongruence caused by reticulation. We conducted a distance network analysis (split graphs) on the ITS dataset to represent simultaneous groupings in the data and evolutionary distance between pairs of taxa. We used the neighbor-net (NN) algorithm [69] as implemented in SplitsTree4 v4.15.1 software [70], with the criterion set to uncorrected pairwise (p) distances, excluding constant and non-informative characters and gap sites.

Finally, to represent possible reticulated evolutionary relationships, a phylogenetic network of ptDNA haplotypes was constructed using a statistical parsimony approach [71] with software TCS 1.21 [72]. Gaps were treated as missing data. Loops obtained in the network due to ambiguities were solved following these criteria: 1) tip and interior relationship, i. e. favoring the union with the innermost haplotype, and 2) geographical location, i. e. favoring the union with the geographically closest haplotype [73].

### Divergence time estimates

Divergence times were estimated using the ITS dataset, which presented better resolution and provided greater statistical support for deep nodes than ptDNA. In addition to the two outgroup species from the *Jacea-Phrygia* group, 11 more outgroup species were added to the matrix used for phylogenetic analysis (S1 Table). We performed dating analyses with BEAST v1.8.3 [74] using a relaxed molecular clock [75]. In view of the lack of fossils from subgenus *Centaurea*, we used two secondary calibration points from a phylogeny of tribe Cardueae, in which six fossils were used as external calibration points [22]. The monophyly of the two outgroup clades was constrained (see nodes 101 and 107; Appendix 3 in Barres et al. [22]) setting a normal prior distribution. A third calibration point, the split between *Centaurea* and the remaining outgroup genera (*Plectocephalus* D. Don., *Psephellus* Cass. and *Rhaponticoides* Vaill.), was established at 6 Ma using the upper limit of the stratigraphic interval of a *Cyanus* pollen type belonging to *Centaurea* [76, 77] choosing a log-normal distribution with an offset associated with the initial fossil age (6 Ma) and a standard deviation of 1.

We performed four preliminary analyses under Yule [78] and birth-death speciation [79] models, and two different relaxed uncorrelated distributions, exponential and log-normal clocks. The MCMC chains were run for 20 million generations, saving one out of 1000 trees using the CIPRES Science Gateway [80]. The Marginal Likelihood Estimators for the four scenarios (combination of Yules and birth-death with exponential and log-normal) were estimated using Path Sampling and Stepping Stone Sampling as previously conducted [81, 82]. We selected the uncorrelated log-normal clock with birth-death speciation model (BD Log) using the Bayes Factor (BF) values (Table 2). Four independent Bayesian MCMC chains were run under the log-normal BD model for 20 million generations, saving one out of 1000 trees. Convergence of the chains and effective sample sizes (ESS>200) were verified with the Tracer package [64] for each individual MCMC additional run. The 25% of the first sampled trees were removed as burn-in, and the resulting trees were combined using LogCombiner. Finally, a maximum clade credibility (MCC) tree was generated using TreeAnnotator software. The resulting MCC tree was transformed into a cladogram using FigTree 1.4.3 [65].

**Table 2:** Estimated BF and MLE values for the four scenarios considered, Yule and Birth-Death, log-normal and exponential clocks using Path Sampling (PS) and Stepping Stone Sampling (SSS). * indicates the selected model. Y = Yule speciation. BD = Birth and Death speciation. Log = log-normal model. Ex = Exponential model.

Speciation and model	2log(BF)	PS	SSS
Y Log	21,6	–4125.1	–4.125.6
Y Ex	12,0	–4120.3	–4.120.3
BD Ex	1,8	–4.115.2	–4.115.7
BD Log	*	–4114.3	–4.114.8

## Results

### nrDNA and ptDNA datasets

The nuclear aligned matrix (ITS) consisted of 85 sequences of 636 bp and 44 parsimony-informative characters for phylogenetic and networking analysis. The ITS matrix for divergence time estimate analysis included 96 sequences of 641 bp (11 additional outgroups). The ptDNA matrix (*rpL16* intron, *rpl32-trnL*^*(UAG)*^, *rps4-trnT-trnL*, *ycf3-trnS*, *trnG*) consisted of 124 sequences of 4291 bp for ptDNA parsimony network analysis, 64 sequences of 4195 bp and 60 informative polymorphic sites for phylogenetic reconstruction (the matrix was reduced by removing redundant sequences of shared haplotypes within the same population). All alignments are available as supplementary material (S1 Appendix).

### nrDNA analyses

**Fig 2:**
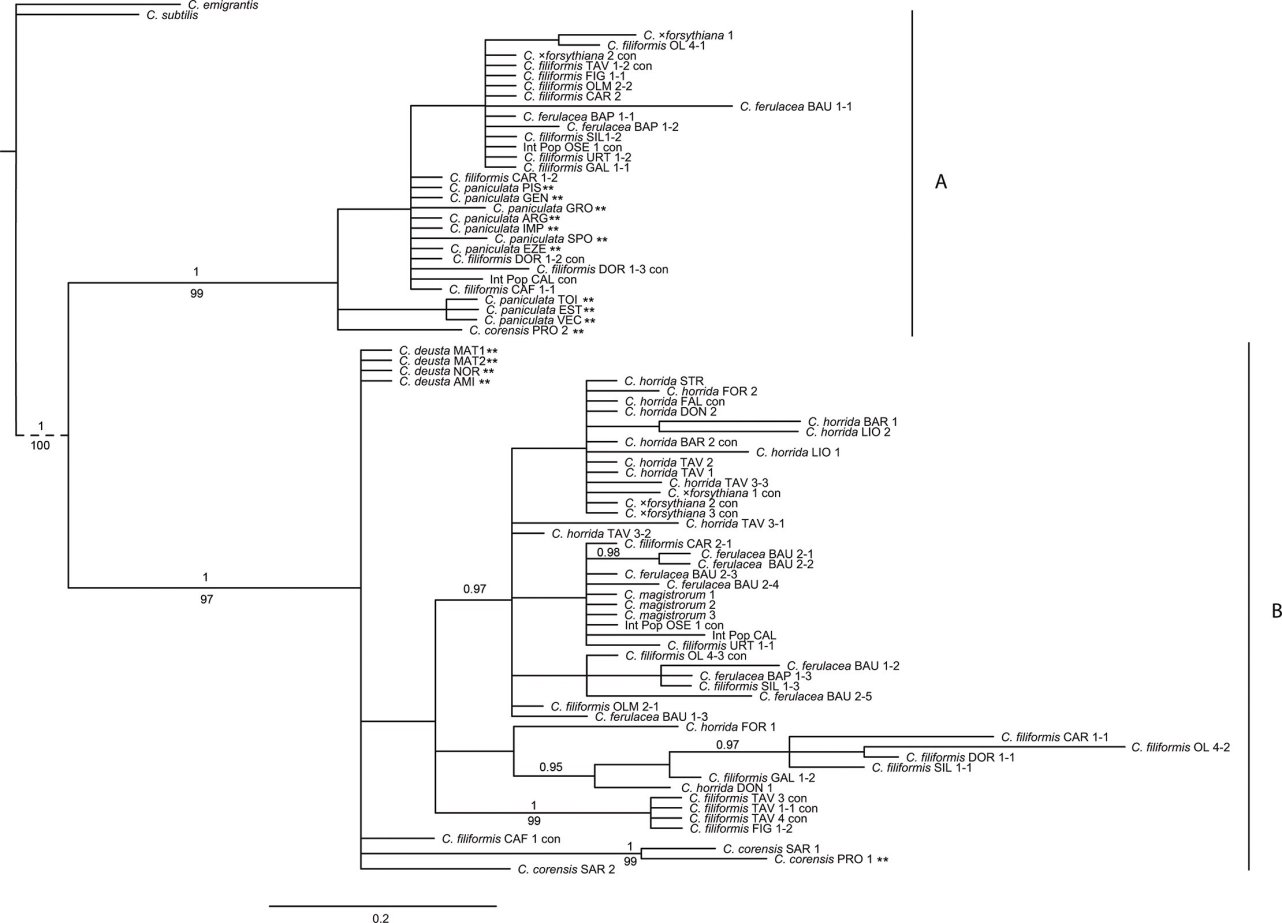
**The 50% majority-rule consensus tree of 5,544 tree obtained by Bayesian analysis of the ITS dataset, indicating supported clades (A and B).** Numbers occurring above branches are posterior probabilities (only PP values higher than 0.95 are considered), whereas bootstrap values occurring beneath branches (only bootstrap values higher than 70% are shown). Capital letters following the names of species correspond to population codes (see S1 Table). The first number identifies the individual, whereas the second, identifies the number of the cloned sequence; con = consensus sequence. ** identifies species and/or populations from the mainland. Numerical results for the maximum parsimony analysis (non-informative characters excluded) are: tree length = 68, CI = 0.4273, RI = 0.8842, and HI = 0.5727. Abbreviations: CI, consistency index; RI, retention index; HI, homoplasy index.

**Fig 3:**
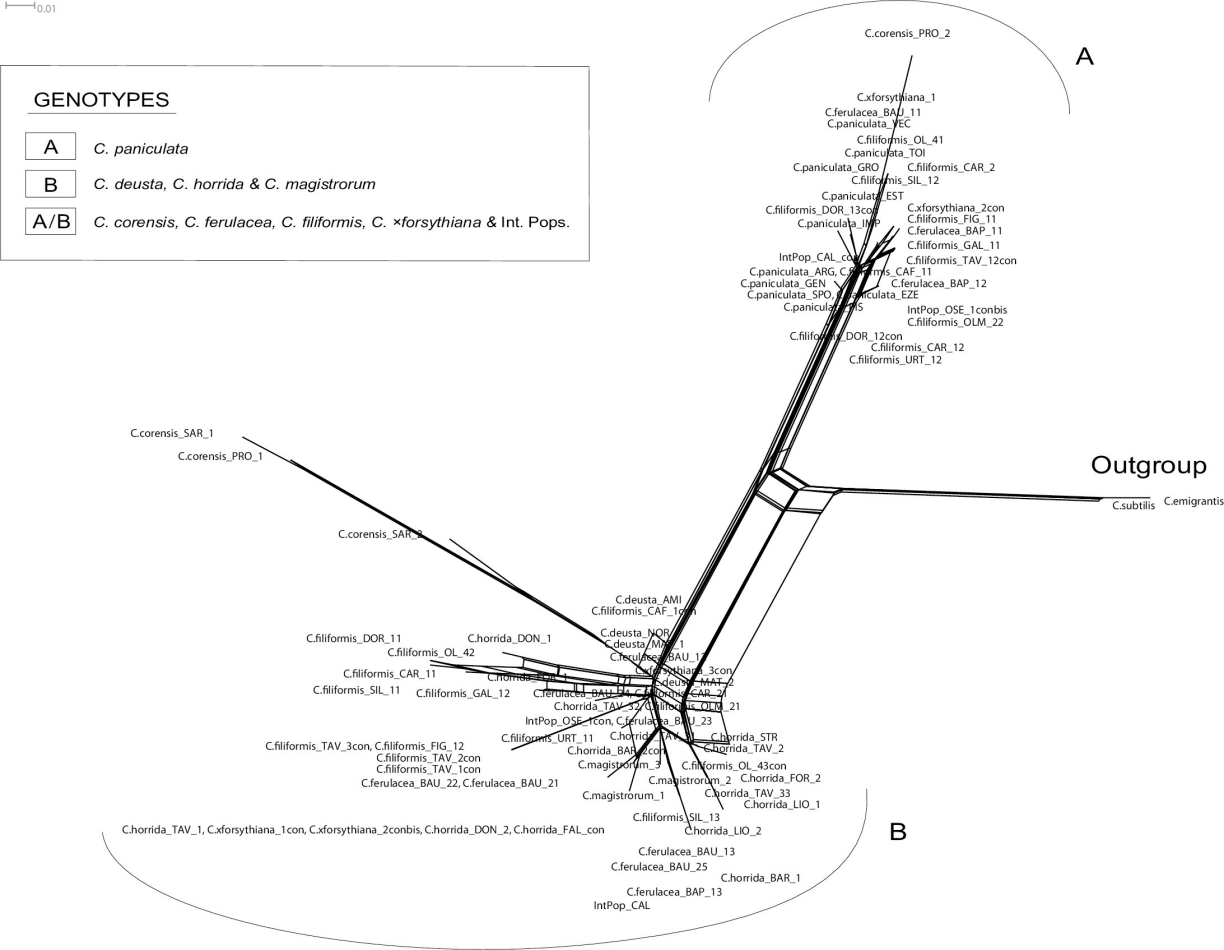
Split graphs based on uncorrected p-distances of the ITS dataset (non-informative, constant characters, and gaps excluded). Capital letters following the names of species correspond to population codes (see S1 Table). The first number identifies the individual, whereas the second identifies the number of the cloned sequence; con = consensus sequence.

Two major clades were defined in the ITS Bayesian and MP phylogenetic trees (Fig 2): Clade A (PP = 1.0, BS = 99%) and Clade B (PP = 1.0, BS = 97%). As revealed by NN analysis (Fig 3), clades A and B corresponded to two different ribotypes. *Centaurea paniculata* was characterized by ribotype A, whereas *C*. *corensis* from Sardinia, *C*. *deusta*, *C*. *horrida* and *C*. *magistrorum* possessed a genome characterized by ribotype B. *Centaurea corensis* from Procida, *C*. *filiformis*, *C*. *ferulacea* (including intermediate populations) and *C*. ×*forsythiana* presented both ribotypes (A and B; Fig 3).

### Plastid DNA analyses

Statistical parsimony analysis of ptDNA revealed 53 haplotypes. The parsimony network (Fig 1B) yielded a complex topology, whereas at least six lineages or haplogroups could be defined. The first (Fig 1B, red) contained individuals belonging to *C*. *filiformis*, *C*. *magistrorum* and *C*. *paniculata*, as well as the population from Monte Oseli (OSE) that was an intermediate between *C*. *filiformis* and *C*. *ferulacea*. A second haplogroup was defined as one that formed exclusively by *C*. *paniculata* individuals (Fig 1B, green). The third haplogroup (Fig 1B, blue) was formed by *C*. *horrida*, *C*. *filiformis* individuals from Tavolara islet (TAV), *C*. *corensis* from Procida Island (PRO), and individuals of *C*. *paniculata* and *C*. *deusta*. The fourth haplogroup (Fig 1B, yellow) was formed by *C*. *corensis* from Sardinia (SAR) and some individuals of *C*. *paniculata*. The fifth haplogroup (Fig 1B, black) was comprised of *C*. *ferulacea*, some individuals of *C*. *filiformis* from Cala Fuili (CAF), the population of *C*. *filiformis* from Cala Sisine, which was intermediate between both species (CAL), and some individuals of *C*. *deusta*. Finally, a sixth haplogroup (Fig 1B, white) was formed exclusively by one individual of *C*. *magistrorum*.

**Fig 4:**
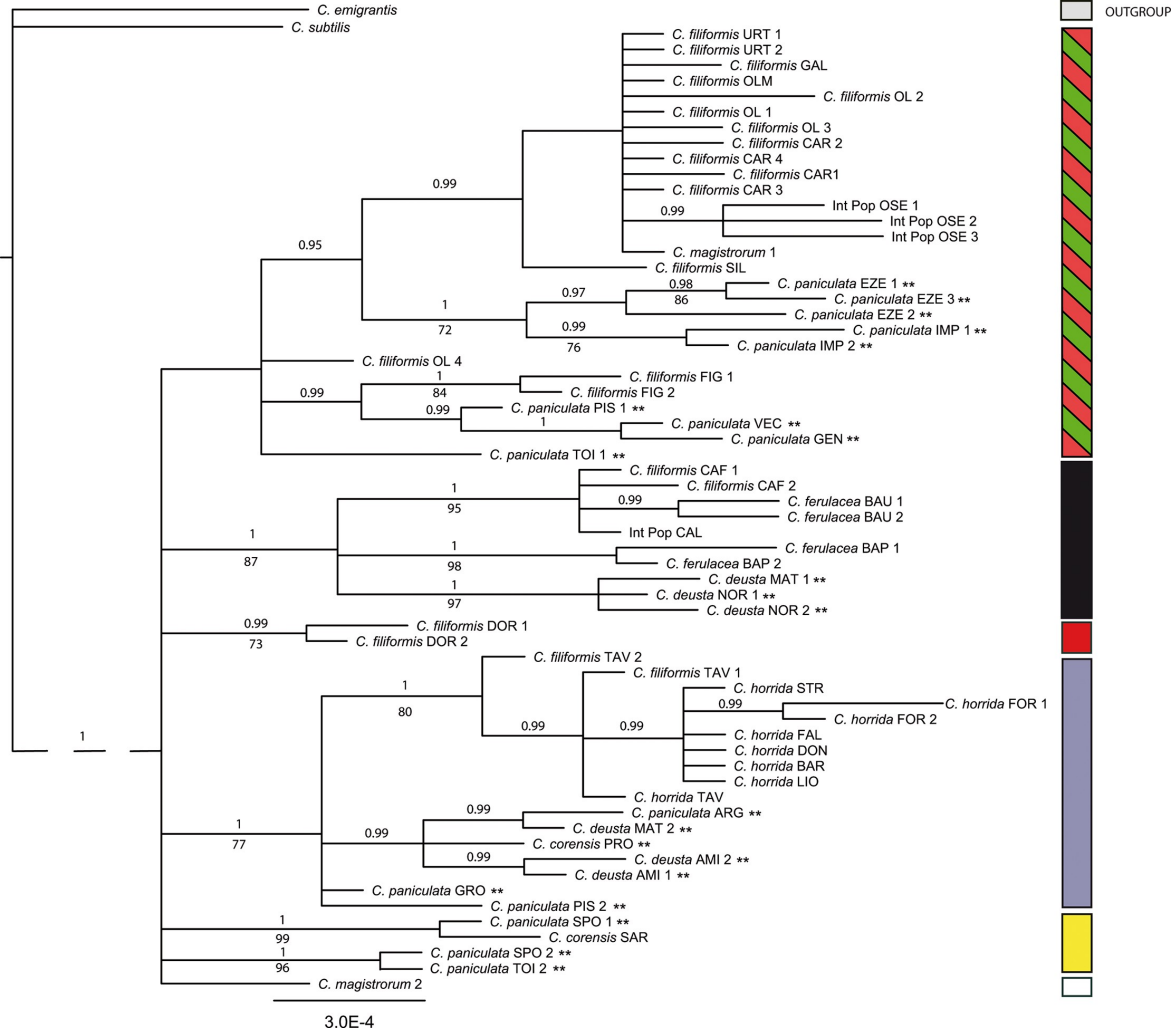
The 50% majority-rule consensus tree of 174,564 tree obtained by Bayesian analysis of the ptDNA dataset, indicating supported clades. Numbers above branches are posterior probabilities (only PP values greater than 0.95 are considered), and numbers below branched are bootstrap values (only BS values greater than 70% are shown). Capital letters following the names of species correspond to population codes (see S1 Table). The number after the code identifies the individual. ** indicate species and/or populations from the mainland. Color bars follow the same codes as for haplotypes in Fig 1B. Numerical results for the maximum parsimony analysis (non-informative characters excluded) are: tree length = 73, CI = 0.7792, RI = 0.9325, and HI = 0.2208. Abbreviations: CI, consistency index; RI, retention index; HI, homoplasy index.

The BI tree (Fig 4) is clearly incongruent with the nrDNA phylogenetic tree (Fig 2). Furthermore, the BI tree is rather similar to the ptDNA network (Fig 1B), even though the red haplogroup (mainly *C*. *filiformis*) and green haplogroup (*C*. *paniculata*) were not retrieved as clearly separated as in the ptDNA network analysis, nor were they statistically supported as a single clade. In the BI tree, there are three supported clades (PP = 0.95 and PP = 0.99; marked in red and red/green in Fig 4), which include individuals of *C*. *filiformis* and *C*. *paniculata*. The clade marked in black (PP = 1.0, BS = 87%) included all the individuals of *C*. *ferulacea*, individuals of *C*. *filiformis* from Cala Fuili (CAF), individuals of an intermediate population from Cala Sisine (CAL), and some individuals of *C*. *deusta*, matching exactly the fifth haplogroup retrieved in the network. The clade marked in blue (PP = 1.0, BS = 77%) fits the third haplogroup in the network and is comprised of, at least, two supported subclades, the first one (PP = 1.0, BS = 80%) including *C*. *horrida* and individuals of *C*. *filiformis* from Tavolara islet, and the second (PP = 0.99) including individuals of *C*. *corensis* from Procida, *C*. *deusta* and *C*. *paniculata*. Although not recovered in a supported clade as in the ptDNA network, the two individual clades (*C*. *paniculata* SPO1-*C*. *corensis* SAR, and *C*. *paniculata* SPO2-TOI2) form the fourth (yellow) haplogroup, which is highlighted in the tree as a putative group to be investigated in future. Finally, the sixth (white) haplogroup recovered in the ptDNA nework and comprised by *C*. *magistrorum*, despite being unresolved, has been also highlighted.

### Divergence time estimates

Results from the dating analyses in BEAST are shown in S1 Fig. The retrieved tree was very similar to tree generated by the nrDNA phylogenetic analysis, with statistical support for clades A and B. Within Clade B, three subclades were also supported. A subclade B1 (node 8) was retrieved as in the ITS BI tree. Likewise, two additional subclades, the first one including two accessions of *C*. *corensis*, one from Procida and one from Sardinia (PP = 1.0), and a second one including two individuals of *C*. *ferulacea* BAU (PP = 1.0), were also recovered as in the ITS BI tree. Age estimations for the deep nodes (Table 3; S1 Fig) indicate a high degree of uncertainty, expressed in the considerable difference between lower and upper 95% Bayesian highest posterior density (HPD). Dating analysis suggests that clades A and B started to diverge between approximately 11.12 and 5.06 Mya (node 5 in Table 3). Clade A is estimated to have started diversifying between 4.77 and 1.26 Mya, whereas Clade B diversified between 6.68 and 2.55 Mya. Within Clade B, the subclade B1, containing most of the accessions of *C*. *horrida*, as well as *C*. *ferulacea*, *C*. *filiformis*, and *C*. *magistrorum*, began diversifying between 3.7 to 1.25 Mya (Clade B1; Table 3; S1 Fig).

**Table 3:** Results of the BEAST dating analyses for the ITS dataset. Node ages are shown only for supported clades.

Clade	Age estimates of different groups (95% HPD lower and upper)
Node 1	19.91 (16.05–23.92)
Node 2	17.8 (14.43–21.38)
Node 3	14.45 (11.25–17.98)
Node 4	12.33 (8.87–16.09)
Node 5	7.95 (5.06–11.12)
Node 6 (Clade A)	2.77 (1.26–4.77)
Node 7 (Clade B)	4.41 (2.55–6.68)
Node 8 (Clade B, subclade B1)	2.30 (1.25–3.70)

## Discussion

### Evolutionary implications

Evolution in *Centaurea* is complex and intriguing, and its study reveals a reticulate pattern of multiple connections [46]. In the case of Sardinia, results from the plastid and nuclear data are not fully congruent (see Figs 2 and 4). Hybridization and lineage sorting of ancestral polymorphisms are the most common explanations for incongruent results, but they are not always easy to differentiate [83, 84]. Hybridization is generally considered to be more probable in recently diversified groups, and lineage sorting of ancestral polymorphisms is more frequent in those that have diversified rapidly [85], but this is of no help in our case: *Centaurea* has diversified recently and has radiated rapidly [83]. In our case, we favor the hybridization hypothesis because intermediate morphological individuals have been described in the Sardinian species [30, 40].

Despite the incongruences caused by introgression, our data allow a hypothetical reconstruction of the relationships of *Centaurea* species in Sardinia. Our plastid results show (Figs 1 and 4) that at least five lineages or haplogroups can be defined on the island: the C. filiformis lineage, the C. horrida lineage, the C. ferulacea lineage, and two additional lineages, which can be partly defined based on plastid data: the C. corensis and C. magistrorum lineages.

#### Centaurea filiformis lineage

As shown in Mameli et al. [30], our nuclear (Figs 2 and 3) and plastid data (Figs 1 and 4) also suggest that *C*. *filiformis* and *C*. *paniculata* are closely related species and we favor the hypothesis of an allopatric speciation event. The red haplotype that is present in one population of *C*. *paniculata* (Fig 1) perhaps indicates sharing of ancestral polymorphism. However, populations of *C*. *paniculata* from Grosseto in continental Italy, which are geographically the closest to Sardinia, did not present plastid haplotypes related for the most part to *C*. *paniculata* or *C*. *filiformis*, but rather to *C*. *horrida*. Nuclear markers, however, revealed only the ribotype A in *C*. *paniculata* and exclusively the ribotype B in *C*. *horrida*. Therefore the presence of a *C*. *horrida-*related haplotype in Grosseto (and partially in Pisa) should be interpreted as one of the frequent cases of hybridization and plastid capture of *C*. *paniculata* in the mainland.

#### Centaurea horrida lineage

Evolutionary relationships within the C. horrida lineage are easily traced because this species is characterized exclusively by ribotype B (Fig 2), and all sampled individuals show a single ptDNA haplotype (Figs 1 and 4). The isolate distribution of *C*. *horrida* in Sardinia, mainly confined to the western part of the island, might explain such a pattern. Actually, *C*. *horrida* hybridizes freely with *C*. *filiformis* giving origin to *C*. *×forsythyana* at the only locality where both occur in eastern Sardinia, namely Tavolara Islet [30]. This introgression suggests a fine example of plastid capture: *C*. *filiformis* from Tavolara possesses the plastid of *C*. *horrida* (Fig 1 see also [30]). *Centaurea horrida* is the only species from Sardinia that does not share its ribotype with *C*. *paniculata*. Its origin probably lies in an unknown species of subsect. *Centaurea*. In support of this hypothesis, Hilpold et al. [33] pointed out that *C*. *horrida* shares a haplotype with *C*. *gymnocarpa* Moris & De Not. from subsect. *Centaurea*.

#### Centaurea ferulacea lineage

The case of *Centaurea ferulacea*, like that of *C*. *filiformis*, relates Sardinian plants to the mainland Italian populations, in particular to *C*. *deusta* (Figs 1–4). Nuclear and plastid DNA analyses are inconclusive (Figs 2 and 4) and relationships are blurred, but plastid evidence suggests an evolutionary relationship between *C*. *deusta* and *C*. *ferulacea*. Furthermore, the case of *C*. *deusta* is similar to that of *C*. *paniculata*, which we considered above: nuclear markers reveal only ribotype B for *C*. *deusta*, and unrelated ptDNA haplotypes for different *C*. *deusta* individuals, indicating possible introgression with an unidentified mainland species of subsect. *Centaurea*. The haplotype is the same in both events, *C*. *deusta* and *C*. *paniculata* (blue haplotype of Fig 1).

Our plastid results (Fig 1) suggest that *C*. *filiformis* and *C*. *ferulacea* are partly genetically differentiated lineages: one of the haplotypes is more related to *C*. *paniculata* (subsect. *Centaurea*), and the other one is closer to *C*. *deusta* (subsect. *Phalolepis*), in agreement with morphology. The similarity in the habit is striking, probably by convergence because both species grow in fissures of limestone. However, bract appendages, which are the main character in the classification of sect. *Centaurea*, are quite different (see also [40]). Thus, we posit that the subordination of *C*. *ferulacea* to *C*. *filiformis* (e.g. [43, 86, 87]) seems unjustified despite the existence of morphologically intermediate populations (Monte Oseli, OSE, and Cala Sisine, CAL], ellipses in Fig 1). Intermediate individuals from the Monte Oseli population in the north present ptDNA from *C*. *filiformis*, whereas intermediate individuals from Cala Sisine in the south present *C*. *ferulacea* ptDNA. Hybridization is surely bidirectional because the hybrids are probably homoploid, and a “hybrid zone” was already defined on genetic grounds by [40]. Furthermore, individuals clearly identified as *C*. *filiformis* on morphological grounds (Cala Fuili, black circle in Fig 1) present ptDNA from the *C*. *ferulacea* line; this suggests that the coastal strip from Baunei to Cala Fuili may represent the ecological corridor followed by *C*. *ferulacea* in its expansion to the north. In contrast, the *C*. *ferulacea* haplotype seems unable to penetrate internal areas in hilly and mountainous regions.

#### Centaurea corensis lineage

Besides the three lineages formed by the most widespread endemic *Centaurea* from Sardinia, a fourth lineage, which is difficult to interpret, has to be considered: the C. corensis lineage (Fig 1). Previous studies have suggested that *C*. *corensis* is a tetraploid hybrid originating on Procida Island (Naples) that was carried to Sardinia by anthropic dispersion [37]. The hypothesis of a long distance, perhaps anthropic, dispersal is supported by our results because *C*. *corensis* from Sardinia presents an exclusive haplotype (yellow haplotype; Fig 1) that is not present in other species from Sardinia and is not derived from other haplotypes present on the island. This haplotype has also been found in two populations of *C*. *paniculata* from southeastern France, and the occurrence of shared ancestral polymorphisms is also a possible explanation, despite being clearly morphologically unrelated. Conversely, the origin of this species in Procida is not supported by our results: the haplotype found in *C*. *corensis* from Procida differed from the haplotype found in *C*. *corensis* from Sardinia. The Sardinian population belongs in the yellow lineage, whereas the population from Procida has a haplotype from the blue line (Fig 1). The possibility of *C*. *corensis* being an allopolyploid with multiple origins would perhaps explain the observed differences. However, elucidating the origin of *C*. *corensis* would require additional studies involving more comprehensive sampling.

#### Centaurea magistrorum lineage

The fifth *Centaurea* species from Sardinia, *C*. *magistrorum*, also shows a complex evolutionary history. We found two ptDNA haplotypes of different origins in the only known population of this species (Fig 1). The most widespread haplotype, which is present in five out of six individuals, corresponds to the main haplotype of *C*. *filiformis*. The second haplotype, found in only one individual, presents no relationship to any haplotype of any other Sardinian species. With regard to nuclear DNA, *C*. *magistrorum* is nested in Clade B together with *C*. *horrida*, *C*. *ferulacea*, and *C*. *deusta*, and representatives of all populations of *C*. *filiformis* (PP = 1.0; BS = 97%; Fig 2). Cloning efforts in *C*. *magistrorum* failed to reveal any ITS copy from Clade A (*C*. *paniculata* complex, including *C*. *filiformis*), which makes it difficult to draw conclusions other than that *Centaurea magistrorum* is surely of hybrid origin. One of its parental species is possibly *C*. *filiformis*, which is morphologically close; however, the identity of both parents remains to be investigated.

### Biogeographic implications

The presence of five lineages of section *Centaurea* on Sardinia (Fig 1) raises the question of how many colonization events have led to the present distribution of the section on the island. The divergence time estimation results (Table 3; S1 Fig) establish the divergence of *Centaurea* sect. *Centaurea* (including, among others, Sardinian *C*. *ferulacea*, *C*. *filiformis*, *C*. *horrida*, and *C*. *magistrorum*) between 11.12–5.06 Mya (Node 5; S1 Fig). Thus, the migration of ancestors from the mainland to Sardinia could have occurred during the Messinian Salinity Crisis (5.96–5.33 Mya [88, 89]). By this time, Sardinia and Corsica were in contact to each other and to northern Italy [7, 10,11], and the re-opening of the Tyrrhenian Sea had only just begun: a link between Sardinia and central Italy via the Calabrian plate was possible at this point [8]. Long distance dispersal cannot be ruled out, but this seems to fit better with the diversification of Clade A (4.77 to 1.26 Mya) than to that of Clade B (6.68 to 2.55 Mya). In this context, two different colonization routes are possible: the first, a northern route from mainland Europe to Sardinia, and the second, a southern route from North Africa to mainland Italy, and finally to Sardinia via the Calabrian plate.

The northern route is considered a possible colonization route for other taxa [90], and it is supported by the affinity of the Corso-Sardinian flora with that of the Tuscan islands. This affinity led to the definition of a Corsican-Sardinian biogeographical province that included the Tuscan Archipelago [5]. In contrast, mainland Italy is the main source to this archipelago’s flora [91], as demonstrated also for other organisms [92]. The shared haplotype between *C*. *filiformis* from Sardinia and a single Ligurian population of *C*. *paniculata* may support the hypothesis that colonization occurred along the northern route. Subsequent isolation would have led to allopatric speciation when the land bridge was disrupted [8, 11]. However, not a single species of *Centaurea* sect. *Centaurea* occurs in Corsica, other than the usual widespread species [93] and the only report for this group indicates a probable anthropic origin [30]. This absence is difficult to explain and therefore long distance dispersal is highly probable. However, other hypotheses such as local extinction or unsuitable ecological conditions are also possible.

An alternative hypothesis involves a southern colonization route, because the inferred ancestral region of *Centaurea* subgenus *Centaurea* is North West Africa [33]. These authors hypothesized that a double colonization event occurred from North Africa: the first one to the Iberian Peninsula via the Strait of Gibraltar, and a second to the Italian Peninsula via Sicily. In view of the possibility of a land bridge connecting Sardinia and the Calabrian Plate to mainland Italy, as previously suggested [8, 94], colonization of Sardinia from the south cannot be ruled out, and indeed has been proposed for other groups [95]. The plastid haplotype composition of *C*. *ferulacea* and *C*. *horrida* is more consistent with southern colonization because their haplotypes dominate both in south and central Italy.

### Conservation implications

Our findings are significant for the conservation of plant diversity in this Mediterranean biodiversity hotspot [2, 3]. Firstly, *C*. *ferulacea* and *C*. *filiformis* appear to represent distinct entities, which is highly relevant for the protection of *C*. *ferulacea*. Moreover, even if the origin of *C*. *corensis* remains unclear and additional studies are required, our results do not support an origin in Procida and it is plausible that this species may constitute an allopolyploid with multiple origins. Considering that four out of five endemic *Centaurea* taxa from Sardinia are threatened, our results contribute knowledge on the genetic variability found in these species and help in the evaluation of which populations should be preserved. *C*. *horrida* is the only species whose priority level is included in annex II of the EU Habitats Directive, and is considered endangered based on IUCN criteria [96], *C*. *ferulacea* was also assessed as being endangered [68], and *C*. *magistrorum* was assessed as being critically endangered [41], as was *C*. *corensis* [97]. Of those *Centaurea* taxa endemic to Sardinia, only *C*. *filiformis*, the more widespread species, is not threatened.

In conclusion, our work stresses the urgent need to develop a dynamic approach for the conservation of plant diversity, based not only on the distribution of taxa (static approach) but by focusing mainly on understanding and preserving genetic diversity. The role of islands as “melting pots” for genetic diversification of terrestrial vascular plants has already been emphasized for other angiosperms in the Corso-Sardinian system [98–100]. The endemic Sardinian *Centaurea* represents a clear example of the need to focus conservation efforts on populations and gene pools rather than just ‘species’. In this study, we confirm that the origin of *C*. *magistrorum* represents an interesting case of triploid hybridization. Furthermore, we suggest that *C*. *×forsythiana* originated by homoploid hybridization [30], and there is active introgression between *C*. *filiformis* and *C*. *ferulacea*. Because the processes of hybridization and introgression have great evolutionary potential in driving differentiation, speciation, and perhaps extinction, of endemic *Centaurea* taxa from Sardinia, the protection of hybrid zones remains urgent and critical for the conservation of biodiversity [98, 99].

## Supporting information

S1 Fig BEAST tree. (PDF)

S1 Table Origin of plant material. Includes Genbank accessions and ID of haplotypes in the network (Fig 1).
(DOCX)

S1 Appendix nrDNA and ptDNA matrices used for phylogenetic, networking analyses and divergence time estimation analysis. (TXT)
