# Supplementary figures and images for: Islands as a crossroad of evolutionary lineages: A case study of Centaurea sect. Centaurea (Compositae) from Sardinia (Mediterranean Basin)
Source: PLoS One. 2020 Feb 7;15(2):e0228776. doi: 10.1371/journal.pone.0228776 (PMC7006937; doi:10.1371/journal.pone.0228776)

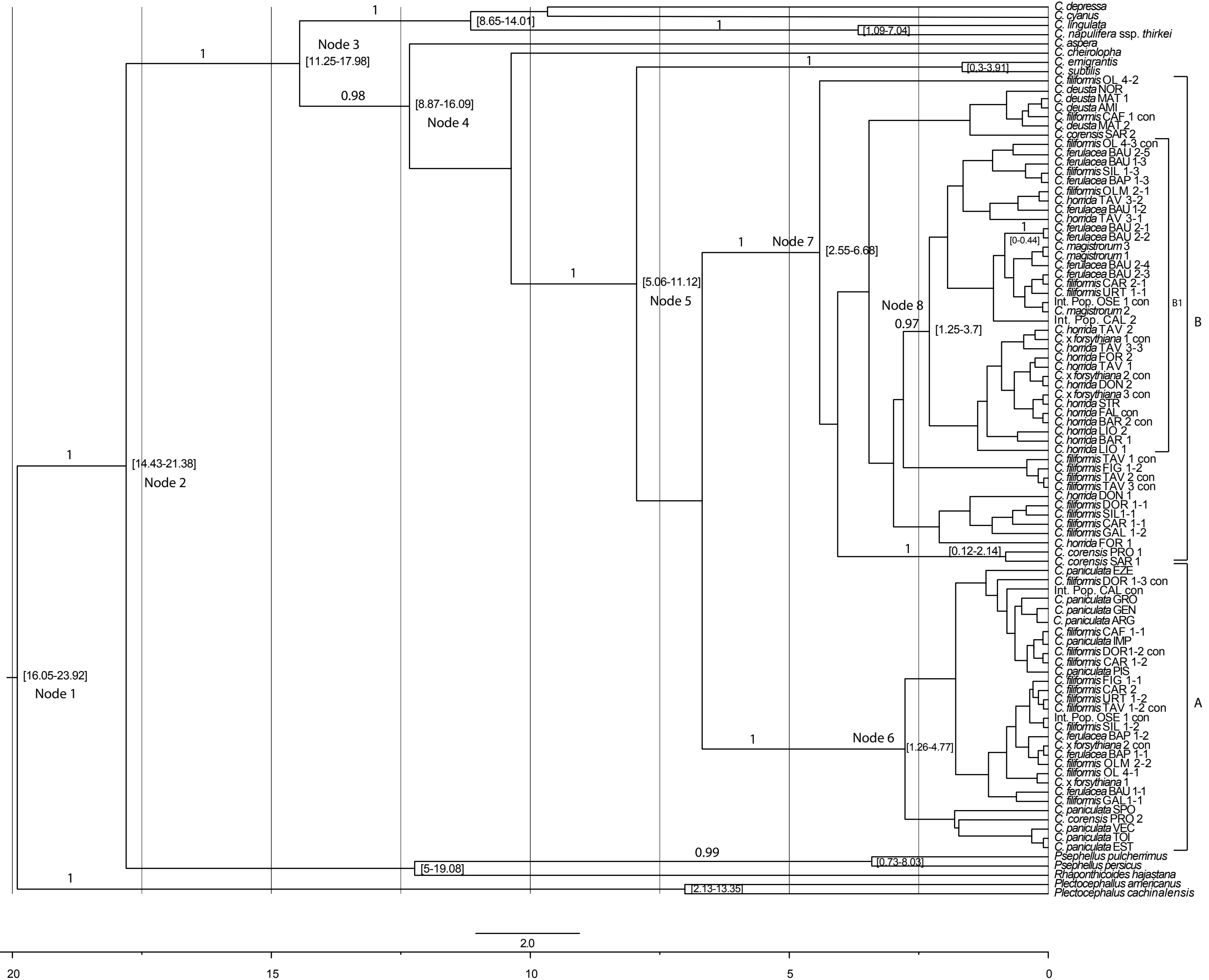

Supplement: S1 Fig — (PDF) [file pone.0228776.s001.pdf]
