# Supplementary material for: Islands as a crossroad of evolutionary lineages: A case study of Centaurea sect. Centaurea (Compositae) from Sardinia (Mediterranean Basin)
Source: PLoS One. 2020 Feb 7;15(2):e0228776. doi: 10.1371/journal.pone.0228776 (PMC7006937; doi:10.1371/journal.pone.0228776)
Supplement: S1 Table — Includes Genbank accessions and ID of haplotypes in the network (Fig 1). (DOCX) [file pone.0228776.s002.docx]

**S1 Table.** **Origin of *Centaurea* material.** Includes Genbank accessions and ID of haplotypes in the network (Fig 1).

| Population code | Location | | Geographic coordinates | Altitude  (m) | | Individuals per population | Haplotype ID | Genbank Accession No. | | | | |  |
| --- | --- | --- | --- | --- | --- | --- | --- | --- | --- | --- | --- | --- | --- |
|  |  |  |  |  |  |  |  | ITS | *rpl16* | *trnG* | *trnT* | *ycf3-trnS* | *rpl32-trnL^(UAG)^* |
| *C. corensis* Vals. & Filigh. | | | | | | | | | | | | | |
| SAR | Italy, Sardinia: Ossi, Scala di Gioca | | 40º41’49”N 8º35’11”E | 320 | | 6 | 41 | KF032426  KF032427 | MH627661  MH627662  MH627663  MH627664  MH627665  MH627666 | MH627829  MH627830  MH627831  MH627832  MH627833  MH627834 | MH627913  MH627914  MH627915  MH627916  MH627917  MH627918 | MH627577  MH627578  MH627579  MH627580  MH627581  MH627582 | MH627745  MH627746  MH627747  MH627748  MH627749  MH627750 |
| PRO | Italy, Naples: Isola di Procida, Terra Murata | | 40º45’42”N  14º2’7”E | 60 | | 5 | 33 | KF032428  KF032431 | MH627667  MH627668  MH627669  MH627670  MH627671 | MH627835  MH627836  MH627837  MH627838  MH627839 | MH627919  MH627920  MH627921  MH627922  MH627923 | MH627583  MH627584  MH627585  MH627586  MH627587 | MH627751  MH627752  MH627753  MH627754  MH627755 |
| *C. deusta* Ten. | | | | | | | | | | | | | |
| AMI | Italy, Toscana: Grosseto, Mt. Amiata, about 2 km E of the Castel del Piano | | 42º53'25''N 11º34'7''E | 850 | | 3 | 34, 35 | MH629979 | MH627684  MH627685  MH627686 | MH627852  MH627853  MH627854 | MH627936  MH627937  MH627938 | MH627600  MH627601  MH627602 | MH627768  MH627769  MH627770 |
| MAT | Italy, Campania: N of San Gregorio Matese | | 41º23'15''N 14º23'11''E | 880 | | 3 | 36, 37, 51 | MN598654  MH629977 | MH627678  MH627679  MH627680 | MH627846  MH627847  MH627848 | MH627930  MH627931  MH627932 | MH627594  MH627595  MH627596 | MH627762  MH627763  MH627764 |
| NOR | Italy, Umbria: hill to 4 km SE Norcia, along road to Marche | | 42º46'3''N 13º8'5''E | 820 | | 3 | 50, 52, 53 | MH629978 | MH627681  MH627682  MH627683 | MH627849  MH627850  MH627851 | MH627933  MH627934  MH627935 | MH627597  MH627598  MH627599 | MH627765  MH627766  MH627767 |
| *C. ferulacea* Martelli | | | | | | | | | | | | | |
| BAU | Italy, Sardinia: Baunei | | 40°02'07.58"N; 9°39'48.68"E | 580 | | 4 | 45, 46, 47 | MH629980  MH629981  MH629982  MH629986  MH629987  MH629988  MH629989  MH629990 | MH627621  MH627622  MH627623  MH627624 | MH627789  MH627790  MH627791  MH627792 | MH627873  MH627874  MH627875  MH627876 | MH627537  MH627538  MH627539  MH627540 | MH627705  MH627706  MH627707  MH627708 |
| BAP | Italy, Sardinia: Monte Scoine-Oro | | 40°00'40.58"N; 9°40'52.40"E | 570 | | 3 | 48, 49 | MH629983  MH629984  MH629985 | MH627625  MH627626  MH627627 | MH627793  MH627794  MH627795 | MH627877  MH627878  MH627879 | MH627541  MH627542  MH627543 | MH627709  MH627710  MH627711 |
| *C. filiformis*Viv. | | | | | | | | | | | | | |
| OL | Italy, Sardinia: Oliena, Sos Prados | | 40°15'22.66"N 9°25'32.11"E | 1196 | | 5 | 7, 8, 9, 12 | KF630455  KF630456  KF630457 | MH921493  MH921494  MH921495  MH921496  MH921497 | KF630584 KF630585 KF630586 KF630587 KF630588 | KF630496 KF630497 KF630498 KF630499 KF630500 | KF630540 KF630541 KF630542 KF630543 KF630544 | MH921533  MH921534  MH921535  MH921536  MH921537 |
| OLM | Italy, Sardinia: Oliena, Monte Maccione | | 40°15'50.83"N 9°24'57.21"E | 665 | | 3 | 9 | KF630458  KF630459 | MH921498  MH921499  MH921500 | KF630589 KF630590 KF630591 | KF630501 KF630502 KF630503 | KF630545 KF630546 KF630547 | MH921538  MH921539  MH921540 |
| CAR | Italy, Sardinia: Cala Cartoe | | 40°19'26.68"N 9°39'45.48"E | 41 | | 6 | 1, 2, 9 | KF630451  KF630452  KF630453  KF630454 | MH921487  MH921488  MH921489  MH921490  MH921491  MH921492 | KF630578 KF630579 KF630580 KF630581 KF630582 KF630583 | KF630490 KF630491 KF630492 KF630493 KF630494 KF630495 | KF630534 KF630535 KF630536 KF630537 KF630538 KF630539 | MH921527  MH921528  MH921529  MH921530  MH921531  MH921532 |
| FIG | Italy, Sardinia: Capo Figari | | 41°00'01.86"N 9°39'24.86"E | 163 | | 3 | 15, 16 | KF630449  KF630450 | MH921484  MH921485  MH921486 | KF630575 KF630576 KF630577 | KF630487 KF630488 KF630489 | KF630531 KF630532 KF630533 | MH921524  MH921525  MH921526 |
| TAV | Italy, Sardinia: Tavolara, Cala del Faro | | 40°55'00.16"N 9°43'25.04"E | 0-200 | | 3 | 28, 29 | KF630444  KF630445  KF630446  KF630447 | MH921481  MH921482  MH921483 | KF630571 KF630572 KF630573 KF630574 | KF630483 KF630484 KF630485 KF630486 | KF630527 KF630528 KF630529 KF630530 | MH921521  MH921522  MH921523 |
| GAL | Italy, Sardinia: Galtellì, Monte Tuttavista | | 40°22'29.14"N; 9°38'08.55"E | 520 | | 3 | 6 | MH630008  MH630009 | MH627612  MH627613  MH627614 | MH627780  MH627781  MH627782 | MH627864  MH627865  MH627866 | MH627528  MH627529  MH627530 | MH627696  MH627697  MH627698 |
| DOR | Italy, Sardinia: Dorgali | | 40°17'14.58"N; 9°35'40.20"E | 554 | | 3 | 13, 14 | MH629997  MH629998  MH629999 | MH627606  MH627607  MH627608 | MH627774  MH627775  MH627776 | MH627858  MH627859  MH627860 | MH627522  MH627523  MH627524 | MH627690  MH627691  MH627692 |
| CAF | Italy, Sardinia: Cala Fuili | | 40°15'23.04"N; 9°37'26.05"E | 0 | | 3 | 43, 44 | MH630006  MH630007 | MH627609  MH627610  MH627611 | MH627777  MH627778  MH627779 | MH627861  MH627862  MH627863 | MH627525  MH627526  MH627527 | MH627693  MH627694  MH627695 |
| SIL | Italy, Sardinia: Urzulei, Genna Silana | | 40°06'04.48"N; 9°30'42.26"E | 800 | | 3 | 10 | MH629994  MH629995  MH629996 | MH627615  MH627616  MH627617 | MH627783  MH627784  MH627785 | MH627867  MH627868  MH627869 | MH627531  MH627532  MH627533 | MH627699  MH627700  MH627701 |
| URT | Italy, Sardinia: Urzulei, Pischina Urtaddala | | 40°10'13.07"N; 9°29'25.61"E | 750 | | 3 | 9 | MH630004  MH630005 | MH627618  MH627619  MH627620 | MH627786  MH627787  MH627788 | MH627870  MH627871  MH627872 | MH627534  MH627535  MH627536 | MH627702  MH627703  MH627704 |
| Intermediate populations (*C. filiformis* × *C. ferulacea*) | | | | | | | | | | | |  | |
| CAL | Italy, Sardinia: Cala Sisine | | 40°10'43.82"N; 9°38'07.27"E | 0 | | 2 | 43 | MH630002  MH630003 | MH627630  MH627631 | MH627798  MH627799 | MH627882  MH627883 | MH627546  MH627547 | MH627714  MH627715 |
| OSE | Italy, Sardinia: Monte Oseli | | 40°07'27.24"N; 9°33'46.86"E | 780 | | 3 | 3, 4, 5 | MH630000  MH630001 | MH627628  MH627629  MH627687 | MH627796  MH627797  MH627855 | MH627880  MH627881  MH627939 | MH627544  MH627545  MH627603 | MH627712  MH627713  MH627771 |
| *C. ×forsythiana* Levier | | | | | | | | | | | |  | |
| TAV | Italy, Sardinia: Tavolara, Cala del Faro | | 40°55'10.61"N 9°43'47.82"E | 2 | | 3 | — | KF630434  KF630436  KF630437  KF630439  KF630441 | — | — | — | — | — |
| *C. horrida* Badarò |  | | | | |  |  |  | | | | |  |
| FOR | Italy, Sardinia: Asinara, Fornelli | | 41°07'11.08''N 8°19'03.75''E | 11 | | 3 | 31, 32 | KF630420 KF630421 | MH921504  MH921505  MH921506 | KF630551 KF630552 KF630553 | KF630463 KF630464 KF630465 | KF630507 KF630508 KF630509 | MH921544  MH921545  MH921546 |
| STR | Italy, Sardinia: Asinara, Piano degli Stretti | | 40°59'54.09''N 8°12'56.59''E | 10 | | 3 | 30 | KF630419 | MH921501  MH921502  MH921503 | KF630548 KF630549 KF630550 | KF630460 | KF630504 KF630505 KF630506 | MH921541  MH921542  MH921543 |
|  |  |  |  |  |  |  |  |  |  |  | KF630461 KF630462 |  |  |
| FAL | Italy, Sardinia: Stintino, Capo Falcone | | 40°57'53.20"N 8°12'6.03"E | 34 | | 2 | 30 | KF630422 | MH921507  MH921508 | KF630554 KF630555 | KF630466 KF630467 | KF630510 KF630511 | MH921547  MH921548 |
| DON | Italy, Sardinia: Stintino, Coscia di Donna | | 40°54'2.96"N 8°13'8.81"E | 2 | | 3 | 30 | KF630423 KF630424 | MH921509  MH921510  MH921511 | KF630556 KF630557 KF630558 | KF630468 KF630469 KF630470 | KF630512 KF630513 KF630514 | MH921549  MH921550  MH921551 |
| LIO | Italy, Sardinia: Alghero, Marina di Lioneddu | | 40°37'32.88"N 8° 9'39.44"E | 126 | | 3 | 30 | KF630427 KF630428 | MH921515  MH921516  MH921517 | KF630562 KF630563 KF630564 | KF630474 KF630475 KF630476 | KF630518 KF630519 KF630520 | MH921555  MH921556  MH921557 |
| BAR | Italy, Sardinia: Alghero, Cala Barca | | 40°36'18.64"N 8° 8'59.27"E | 27 | | 3 | 30 | KF630425 KF630426 | MH921512  MH921513  MH921514 | KF630559 KF630560 KF630561 | KF630471 KF630472 KF630473 | KF630515 KF630516 KF630517 | MH921552  MH921553  MH921554 |
| TAV | Italy, Sardinia: Tavolara, Cala del Faro | | 40°53'46.54"N 9°41'14.32"E | 10 | | 3 | 29 | KF630429 KF630430 KF630431 KF630432 KF630433 | MH921518  MH921519  MH921520 | KF630565 KF630566 KF630567 | KF630477 KF630478 KF630479 | KF630521 KF630522 KF630523 | MH921558  MH921559  MH921560 |
| *C. magistrorum* Arrigoni & Camarda | | | | | |  |  |  | | | | |  |
| MAG | Italy, Sardinia: Villagrande Strisaili, Monte Luas | | 39°57'24.0"N 9°30'30.8"E | 850 | | 6 | 9, 39 | MH629991  MH629992  MH629993 | MH627672  MH627673  MH627674  MH627675  MH627676  MH627677 | MH627840  MH627841  MH627842  MH627843  MH627844  MH627845 | MH627924  MH627925  MH627926  MH627927  MH627928  MH627929 | MH627588  MH627589  MH627590  MH627591  MH627592  MH627593 | MH627756  MH627757  MH627758  MH627759  MH627760  MH627761 |
| *^[[1]](#footnote-1)^C. paniculata* L. complex (including several species; species names in the original publication) | | | | | | | | | | | |  | |
| EZE | France, Alpes-Maritimes: along the road from Èze-Bord-de-Mer to Beaulieu-sur-Mer, Col d’Èze | | 43º42'59''N 7º21'2''E | 5 | | 3 | 20, 21, 22 | KJ666042 | MH627632  MH627633  MH627634 | MH627800  MH627801  MH627802 | MH627884  MH627885  MH627886 | MH627548  MH627549  MH627550 | MH627716  MH627717  MH627718 |
| EST | France, Alpes-Maritimes: Forêt domainale de l'Esterell, road to Col des Trois Termes | | 43º31'13''N 6º53'51''E | 100 | | 3 | 23 | KJ666064 | MH627658  MH627659  MH627660 | MH627826  MH627827  MH627828 | MH627910  MH627911  MH627912 | MH627574  MH627575  MH627576 | MH627742  MH627743  MH627744 |
| IMP | Italy, Imperia: Ventimiglia to Porra, about 1.5 km N of Roverino | | 43°49'2"N 7°35'31"E | 30 | | 3 | 24, 25 | KJ666062 | MH627655  MH627656  MH627657 | MH627823  MH627824  MH627825 | MH627907  MH627908  MH627909 | MH627571  MH627572  MH627573 | MH627739  MH627740  MH627741 |
| SPO | Italy, Savona: 2 km W of the centre of Spotorno | | 44°13'46"N 8°24.2"E | 100 | | 2 | 40, 42 | KF032416 | MH627635  MH627636 | MH627803  MH627804 | MH627887  MH627888 | MH627551  MH627552 | MH627719  MH627720 |
| TOI | Italy, Savona: Toirano to Balestrino | | 44º7'22''N 7º11'35'E | 100 | | 3 | 11, 42 | KJ666041 | MH627652  MH627653  MH627654 | MH627820  MH627821  MH627822 | MH627904  MH627905  MH627906 | MH627568  MH627569  MH627570 | MH627736  MH627737  MH627738 |
| GEN | Italy, Genova: Chiavari | | 44º19'31''N 9º19'2''E | 50 | | 3 | 19 | KJ665912 | MH627649  MH627650  MH627651 | MH627817  MH627818  MH627819 | MH627901  MH627902  MH627903 | MH627565  MH627566  MH627567 | MH627733  MH627734  MH627735 |
| PIS | Italy, Pisa: Migliarino, river’s Serchio estuary | | 43º47'7''N 10º16'8''E | 2 | | 3 | 17, 27 | KJ666061 | MH627643  MH627644  MH627645 | MH627811  MH627812  MH627813 | MH627895  MH627896  MH627897 | MH627559  MH627560  MH627561 | MH627727  MH627728  MH627729 |
| VEC | Italy, Pisa: Marina di Vecchiano | | 43º47'43''N 10º16'0''E | 2 | | 3 | 18 | KJ665919 | MH627646  MH627647  MH627648 | MH627814  MH627815  MH627816 | MH627898  MH627899  MH627900 | MH627562  MH627563  MH627564 | MH627730  MH627731  MH627732 |
| ARG | Italy, Grosseto: Argentario, Mascherino to Padri Passionati | | 42º25'5''N 11º9'36''E | 230 | | 3 | 38 | KJ665914 | MH627637  MH627638  MH627639 | MH627805  MH627806  MH627807 | MH627889  MH627890  MH627891 | MH627553  MH627554  MH627555 | MH627721  MH627722  MH627723 |
| GRO | Italy, Grosseto: Valpiana to Lago dell’Accesa | | 42º59'52''N 10º52'52''E | 200 | | 3 | 26 | KJ665913 | MH627640  MH627641  MH627642 | MH627808  MH627809  MH627810 | MH627892  MH627893  MH627894 | MH627556  MH627557  MH627558 | MH627724  MH627725  MH627726 |
| *Jacea-Phrygia* group Outgroup |  | | | | |  |  |  | | | | |  |
| *C. emigrantis* Bubani | Spain, Lleida: Llimiana, along the road LV-9121 to Llimiana | | 42°03'37.7"N 0°53'37.5"E | 400 | | 1 | — | MH629976 | MH627604 | MH627772 | MH627856 | MH627520 | MH627688 |
| *C. subtilis* Bertol. | Italy, Gargano peninsula: 0.5 km N of Madonne delle Grazie | | 41°42'37.0"N 15°56'46.9"E | 280 | | 1 | — | FJ572057 | MH627605 | MH627773 | MH627857 | MH627521 | MH627689 |
| Divergence time estimation analysis outgroups’ | | | Genbank Accession No. | | Reference | | | | | | | | |
| *Centaurea aspera* L. | |  | DQ319086 | | Garcia-Jacas N, Uysal T, Romashchenko KY, Suárez-Santiago VN, Ertuğrul K, Susanna A. *Centaurea* revisited: a molecular survey of the *Centaurea jacea* group. Ann Bot. 2006; 98: 741–753 | | | | | | | | |
| *Centaurea cheirolopha* (Fenzl.) Wagenitz | |  | DQ319101 | | Garcia-Jacas N, Uysal T, Romashchenko KY, Suárez-Santiago VN, Ertuğrul K, Susanna A. *Centaurea* revisited: a molecular survey of the *Centaurea jacea* group. Ann Bot. 2006; 98: 741–753 | | | | | | | | |
| *Centaurea cyanus* L. | |  | AY826254 | | Susanna A, Garcia-Jacas N, Hidalgo O, Vilatersana R, GarnatjeT. The Cardueae (Compositae) revisited: Insights from ITS, trnL-trnF  and matK nuclear and chloroplast DNA analysis. Ann Mo Bot Gard. 2006; 93: 150–171 | | | | | | | | |
| *Centaurea depressa* M. Bieb. | |  | AY826255 | | Susanna A, Garcia-Jacas N, Hidalgo O, Vilatersana R, GarnatjeT. The Cardueae (Compositae) revisited: Insights from ITS, *trnL-trnF*  and *matK* nuclear and chloroplast DNA analysis. Ann Mo Bot Gard. 2006; 93: 150–171 | | | | | | | | |
| *Centaurea lingulata* Lag. | |  | AY826258 | | Susanna A, Garcia-Jacas N, Hidalgo O, Vilatersana R, GarnatjeT. The Cardueae (Compositae) revisited: Insights from ITS, *trnL-trnF*  and *matK* nuclear and chloroplast DNA analysis. Ann Mo Bot Gard. 2006; 93: 150–171 | | | | | | | | |
| *Centaurea napulifera* Rochel | |  | DQ319136 | | Garcia-Jacas N, Uysal T, Romashchenko KY, Suárez-Santiago VN, Ertuğrul K, Susanna A. *Centaurea* revisited: a molecular survey of the *Centaurea jacea* group. Ann Bot. 2006; 98: 741–753 | | | | | | | | |
| *Plectocephalus americanus*(Nutt.) D. Don. | | | JF754817 | | Susanna A, Galbany-Casals M, Romaschenko K, Barres L, Martin J, Garcia-Jacas N. Lessons from *Plectocephalus* (Compositae, Cardueae-Centaureinae): ITS disorientation in annuals and Beringian dispersal as revealed by molecular analyses. Ann. Bot. 2011; 108: 263–277 | | | | | | | | |
| *Plectocephalus cachinalensis*(Phil.) N. Garcia & Susanna | | | JF754804 | | Susanna A, Galbany-Casals M, Romaschenko K, Barres L, Martin J, Garcia-Jacas N. Lessons from *Plectocephalus* (Compositae, Cardueae-Centaureinae): ITS disorientation in annuals and Beringian dispersal as revealed by molecular analyses. Ann. Bot. 2011; 108: 263–277 | | | | | | | | |
| *Psephellus persicus*(DC) Wagenitz | | | AY826316 | | Hidalgo O, Garcia-Jacas N, Garnatje T, Susanna A. Phylogeny of *Rhaponticum* (Asteraceae, Cardueae-Centaureinae) and related genera inferred from nuclear and chloroplast DNA sequence data: taxonomic and biogeographic implications. Ann. Bot. 2006; 97: 705–714 | | | | | | | | |
| *Psephellus pulcherrimus* (Willd.) Wagenitz | | | AY826317 | | Hidalgo O, Garcia-Jacas N, Garnatje T, Susanna A. Phylogeny of Rhaponticum (Asteraceae, Cardueae-Centaureinae) and related genera inferred from nuclear and chloroplast DNA sequence data: taxonomic and biogeographic implications. Ann. Bot. 2006; 97: 705–714 | | | | | | | | |
| *Rhaponticoides hajastana* (Tzevelev) M. V. Agab & Greuter | | | AY826235 | | Susanna A, Garcia-Jacas N, Hidalgo O, Vilatersana R, GarnatjeT. The Cardueae (Compositae) revisited: Insights from ITS, *trnL-trnF*  and *matK* nuclear and chloroplast DNA analysis. Ann Mo Bot Gard. 2006; 93: 150–171 | | | | | | | | |

1. [↑](#footnote-ref-1)
